# Developing quality indicators for the care of HIV-infected pregnant women in the Dutch Caribbean

**DOI:** 10.1186/1742-6405-8-32

**Published:** 2011-09-22

**Authors:** Hillegonda S Hermanides, Lonneke A van Vught, Ralph Voigt, Fred D Muskiet, Aimée Durand, Gerard van Osch, Sharline Koolman-Wever, Isaac Gerstenbluth, Colette Smit, Ashley J Duits

**Affiliations:** 1 Red Cross Blood Bank Foundation, Willemstad, Curaçao; 2 St Elisabeth Hospital, Willemstad, Curaçao; 3 Ofisina Van Osch, Union Road 139e, Cole Bay, St Maarten; 4 Services of Contagious Diseases, Department of Public Health of Aruba, Oranjestad, Aruba; 5 Epidemiology and Research Unit, Medical and Public Health Service of Curaçao, Willemstad, Curaçao; 6 Stichting HIV Monitoring (SHM), Amsterdam, The Netherlands

**Keywords:** HIV, Mother-to-Child Transmission, quality indicator, Caribbean

## Abstract

**Background:**

Effective interventions to prevent mother-to-child HIV transmission (PMTCT) exist and when properly applied reduce the risk of vertical HIV transmission. As part of optimizing PMTCT in the Dutch Caribbean we developed a set of valid and applicable indicators in order to assess the quality of care in HIV-infected (pregnant) women and their newborns.

**Methods:**

A multidisciplinary expert panel of 19 experts reviewed and prioritized recommendations extracted from locally used international PMTCT guidelines according to a 3-step-modified-Delphi procedure. Subsequently, the feasibility, sample size, inter-observer reliability, sensitivity to change and case mixed stability of the potential indicators were tested for a data set of 153 HIV-infected women, 108 pregnancies of HIV-infected women and 79 newborns of HIV-infected women in Aruba, Curaçao and St Maarten from 2000 to 2010.

**Results:**

The panel selected and prioritized 13 potential indicators. Applicability could not be tested for 4 indicators regarding HIV-screening in pregnant women because of lack of data. Four indicators performed satisfactorily for Curaçao ('monitoring CD4-cell count', 'monitoring HIV-RNA levels', 'intrapartum antiretroviral therapy and infant prophylaxis if antepartum antiretroviral therapy was not received', 'scheduled caesarean delivery') and 3 for St Maarten ('monitoring CD4-cell count', 'monitoring HIV-RNA levels', 'discuss and provide combined antiretroviral therapy to all HIV-infected pregnant women') whilst none for Aruba.

**Conclusions:**

A systemic evidence-and consensus-based approach was used to develop quality indicators in 3 Dutch Caribbean settings. The varying results of the applicability testing accentuate the necessity of applicability testing even in, at first, comparable settings.

## Background

Acquired immunodeficiency syndrome (AIDS) is a leading cause of illness and death among women and children in countries with high rates of human immunodeficiency virus (HIV) infection [1]. Mother-To-Child HIV Transmission (MTCT) is by far the most significant route of HIV-infection in children. Several interventions have proven to be effective in reducing MTCT, including elective caesarean delivery [2,3], substitution of breastfeeding [4-6] and access to antiretroviral therapy during pregnancy, labour and post-partum [7]. If properly applied, these interventions reduce the MTCT rates to 2% [8,9].

In the Netherlands Antilles, 1812 HIV-1-cases were reported in 2008, with 83 new cases in 2007. The Dutch Caribbean consists of Aruba and the Netherlands Antilles (Saba, St Eustatia, Bonaire, St Maarten and Curaçao) and has an estimated prevalence of HIV-1-infection of 0.61%-1.05% in the adult population [10]. Forty percent of the registered patients are female and there have been approximately 5 to 10 pregnancies in HIV-infected women annually.

Since 1996 guidelines regarding the prevention of mother-to-child HIV transmission (PMTCT) have been implemented in regular health care systems in the Dutch Caribbean and the annual number of paediatric HIV-cases has dropped dramatically since [10]. However, new paediatric HIV-cases have been reported in recent years. Limited data on the quality of care provided after implementation of the guidelines are available and the question rises as to whether opportunities for the prevention of HIV transmission were missed. Monitoring and evaluating the quality of care in HIV-infected women to achieve PMTCT is important as it can identify strategies to improve the quality of care provided and thereby lead to a better outcome in the prevention of HIV transmission [11]. As part of optimizing the quality of prenatal and delivery care in HIV-infected (pregnant) women in the Dutch Caribbean, this study aims to develop a validated and applicable set of quality indicators to measure the quality of care in HIV-infected (pregnant) women and their newborns in 3 Dutch Caribbean settings; Aruba, Curaçao and St Maarten.

## Methods

### Phase 1: Consensus procedure

Locally used PMTCT guidelines, including guidelines for care of HIV-infected pregnant women, were collected from which a hundred key recommendations were pre-selected by three independent researchers. An extensive literature search was performed using PubMed to identify already existing quality of care indicators for the care of HIV-infected pregnant women. On the basis of the available literature, the level of evidence was graded [12] for each recommendation to determine its scientific soundness or the likelihood that improvement of the quality indicator reflects improvements in quality of care [13]. (Table 1) According to a 3-step-Delphi-approach the group judgement of experts was used to assess the validity of the preselected recommendations [14]. During 3 rating rounds an expert panel rated the preselected recommendations by judging their relevance with regard to effectiveness of the intervention related to PMTCT, the applicability of the recommendation for the current setting, and health care costs [15-17]. The multidisciplinary expert team consisted of 19 experts: 3 paediatricians, 3 gynaecologists, 3 midwifes, 2 general practitioners, 2 epidemiologists, 3 internal medicine specialists, 2 HIV/AIDS programme managers and 1 microbiologist. After the selection and prioritization the recommendations were further developed as potential indicator by defining its numerator and denominator.

**Table 1:** Level of supporting evidence

Level of Supporting evidence	Definition	Example
A1	A good systematic review of studies designed to answer the question of interest.	Systematic review of randomized controlled trials.
A2	One or more rigorous studies designed to answer the question but not formally combined.	Randomized controlled trial.
B	One or more prospective clinical studies that illuminate but do not rigorously answer the question.	Prospective cohort study; unpowered or poor quality randomized controlled trial; or nonrandomized controlled trial.
C	One or more retrospective clinical studies that illuminate but do not rigorously answer the question.	Audit or retrospective case-control study.
D	Formal combination of expert views or other information.	Delphi study; expert opinion; informed consensus.

Data are from (12).

### Phase 2: Applicability test of potential quality indicators

**Figure 1:**
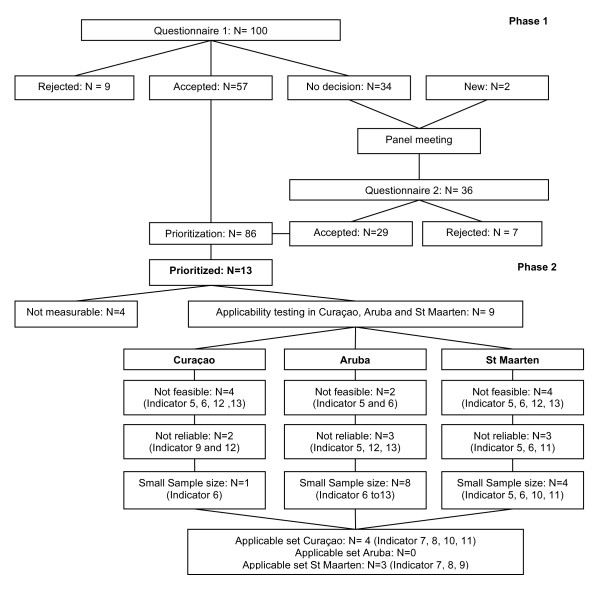
**Flow chart showing the development of quality indicators during the consensus procedure of phase 1 of the study and the applicability testing of phase 2 of the study**.

Before the indicator set is used in a specific setting, its applicability in the chosen practice setting has to be tested. The next step is therefore to provide empirical evidence of the feasibility, sample size, reliability, sensitivity to change and case mix stability of each indicator. (Figure 1) Since national registries of pregnancies are not available in the Dutch Caribbean, the applicability testing of the set of potential indicators was limited to the outpatient clinical setting of the HIV specialists and the clinical setting of the general hospitals in Aruba, Curaçao and St Maarten. Eligible patients included HIV-infected women of childbearing age, HIV-infected pregnant women, and exposed children between January 2000 and January 2010. Data were selected by using clinical data systems of the general hospitals, the outpatient clinic of the gynaecologists, paediatricians, HIV specialists and national registries available at the Public Health Department of each island. In Curaçao, a national electronic registration system (Stichting HIV Monitoring, SHM)[18] was consulted and in Aruba, the national registration database of the Services of Contagious Diseases, Public Health Department was used to select HIV-infected women of childbearing age. In St Maarten, no electronic database was available, therefore no patient selection could be made for indicators regarding HIV-infected women of childbearing age. Non-electronic registrations conducted by health care workers were also consulted in the 3 settings. Excluded from analysis were pregnancies ending before the second trimester, pregnancies ending in abortion with unknown gestation duration, or deliveries abroad.

#### Feasibility

of the indicator was defined as the availability of administrative data required to evaluate the indicator. An indicator was considered to be feasible if the data necessary to score the indicator could be abstracted from the available data for > 70% of the cases [19].

#### Sample size

of the indicator was related to the number of patients to whom the indicator could be applied. Considering the existing literature, the period and the estimated number of patients or events eligible for this study, the research team considered an indicator to be applicable if it could be applied to at least 15 patients or events based on consensus rather than statistical analysis.

#### Inter-rater reliability

refers to the extent in which a measurement of an indicator is reproducible, between observers and between cases. A second investigator rated 10% of all the records in the 3 different medical centres to assess the inter-rater reliability. To assess the agreement between 2 investigators corrected for chance, a Cohen kappa coefficient was calculated. Indicators with a value of κ < 0.60 were considered unreliable [20].

#### Sensitivity to change

was defined as the need to detect changes in the quality of care in order to discriminate between and within subjects hence showing the possibilities for improvement in the present care. Potential indicators with an overall performance score of > 85% were defined as having little room for improvement and were not selected [21].

#### Case mix stability

referred to the need for the correction of certain patient characteristics. The relationship between patient parameters and the indicator result can identify whether there is need for correction for case mix. Indicators that are not case mix stable require comparable patient populations when comparing the quality of care. Patient characteristics possibly influencing the quality of care were defined as: type of health care insurance, age, not born in the Dutch Caribbean and number of previous deliveries. Outcome of the indicator was supposed to be influenced by the patient characteristic if the p < 0.05. Correction of these patient characteristics was performed and analysed if the characteristics were of influence to the outcome of the indicator.

## Results

### Phase 1: Consensus procedure

Of the in total 19 panel members, 15 panellists (79%) completed the questionnaire in the first round, 15 panellists (79%) completed the second round and 10 panellists (53%) were present during the panel meeting. After the first rating round 57 recommendations were rated as potential indicators. (Figure 1) Nine recommendations were considered not-suitable as potential indicators. Thirty-three recommendations were discussed and reformulated during the panel meeting. Two recommendations were added. More than 200 comments were added, encoded and grouped by the research team for discussion during the panel meeting. After the second rating round 28 recommendations were selected as potential indicators and 7 recommendations were rejected. A final set of 13 recommendations was prioritized for which numerators and denominators were defined. (Table 2)

**Table 2:** Applicability of potential quality indicators for the care of HIV-1-infected (pregnant) women and their newborns in Curaçao, Aruba and St Maarten.

Indicator, setting	Sample size, number of patients	Feasibility, % of available data	Inter-rater reliability, κ	Sensitivity to change, %	Case-mix stable
Pregnant women					
1. HIV testing should be done in all pregnant women.	NA	0	NA	NA	NA
2. Pregnant women who decline HIV testing should be encouraged to be tested at subsequent visits.	NA	0	NA	NA	NA
3. Repeat HIV testing if risk factors are present during pregnancy.	NA	0	NA	NA	NA
4. Perform HIV rapid testing if HIV status is unknown at labour.	NA	0	NA	NA	NA
HIV-infected women					
5. Offer preconception counseling and care to HIV-infected women of childbearing potential.					
Total	153	31	0.54	45	Yes
Curaçao	136	18	0.60	35	Yes
Aruba	17	59	< 0.0	83	Yes
St Maarten	NA	0	NA	NA	NA
6. Maximally suppress plasma HIV RNA levels prior to conception in HIV-infected women who wish to get pregnant.					
Total	14	18	0.82	50	Yes
Curaçao	12	15	0.82	50	Yes
Aruba	2	18	1	50	NA
St Maarten	NA	0	NA	NA	NA
HIV-infected pregnant women					
7. Monitor CD4 cell count at the initial visit and at least every 3 months during pregnancy.					
Total	**91**	**97**	**0.92**	**16**	**No^2^**
Curaçao	**54**	**94**	**1**	**18**	**Yes**
Aruba	8	100	1	0	NA
St Maarten	**29**	**100**	**0.67**	**18**	**Yes**
8. Monitor plasma HIV RNA levels at initial visit, 2 to 6 weeks after start antiretroviral therapy, monthly until undetectable, and then at least every 2 months during pregnancy.					
Total	**91**	**81**	**0.92**	**0**	**NA**
Curaçao	**54**	**80**	**0.86**	**0**	**NA**
Aruba	8	100	1.0	0	NA
St Maarten	**29**	**80**	**0.67**	**0**	**NA**
9. Discuss and provide combined antiretroviral prophylaxis to all					
HIV-infected pregnant women, regardless HIV RNA levels.					
Total	91	92	0.57	74	Yes
Curaçao	54	91	0.52	77	Yes
Aruba	8	100	0.67	75	NA
St Maarten	**29**	**93**	**1**	**70**	**Yes**
10. Give intrapartum and infant antiretroviral prophylaxis to all HIV-infected pregnant women who do not receive antepartum antiretroviral therapy.					
Total	**24**	**92**	**0.76**	**0**	**NA**
Curaçao	**16**	**91**	**0.72**	**0**	**NA**
Aruba	2	100	0.67	0	NA
St Maarten	6	93	1	0	NA
11. Perform a cesarean delivery at 38 weeks gestation if HIV RNA levels > 400 copies/mL or unknown.					
Total	**53**	**92**	**0.74**	**49**	**No^3^**
Curaçao	**35**	**96**	**0.93**	**60**	**Yes**
Aruba	7	100	0.60	29	NA
St Maarten	11	83	0.35	27	Yes
12. Counsel HIV-infected pregnant women to avoid breastfeeding.					
Total	91	65	0.06	81	Yes
Curaçao	54	67	-0.29	50	Yes
Aruba	8	100	0	88	NA
St Maarten	29	52	1	93	No^**1**^
Newborn					
13. Continue antiretroviral prophylaxis in the newborn during 4 weeks post partum.					
Total	79	24	0.77	79	Yes
Curaçao	49	24	0.81	50	Yes
Aruba	8	75	0.11	33	NA
St Maarten	22	50	1	93	Yes

The indicators that were applicable in practice are shown in boldface font. NA, not applicable. ^**1 **^Correction for multiparity, ^**2 **^Correction for women not born in Dutch Caribbean, multi-parity and age, ^**3 **^Correction for insurance type.

### Phase 2: Applicability test of potential quality indicators

The applicability test of the set of potential indicators took place in Curaçao, St Maarten and Aruba from January 2010 till April 2010. Four potential indicators selected by the panellists focused primarily on HIV screening in pregnant women with unknown HIV status. However, due to the lack of registration systems for pregnancies in the Dutch Caribbean no data of pregnant women could be retrieved and the applicability of the 4 'screening indicators' could not be tested. The practice setting was limited to HIV-infected (pregnant) women and their newborns on which the other 9 potential indicators could be applied. Inclusion of eligible patients led to a total number of 153 HIV-infected women of child bearing potential (136 in Curaçao, 17 in Aruba, with no data availability for St Maarten), 108 pregnancies of 91 HIV-infected women (54 in Curaçao, 8 in Aruba and 29 in St Maarten) and 79 live born children of HIV-infected women (49 in Curaçao, 8 in Aruba and 22 in St Maarten). Twelve pregnancies were excluded because they ended before the second trimester of gestation (10 in Curaçao, 2 in St Maarten). Five pregnancies were excluded due to an abortion after unknown pregnancy duration (3 in Curaçao, 2 in St Maarten).

#### Feasibility

Indicator 5 ('preconception counselling for all HIV-infected women') had a low feasibility for Curaçao (18% of patients had available data) and moderate feasibility for Aruba (59%). Indicator 6 ('maximally suppress viral load in HIV-infected women who wish to get pregnant') scored low feasibility in Curaçao and Aruba (15% and 17% respectively). In St Maarten feasibility for indicator 5 and indicator 6 could not be assessed, as there was no data set of HIV-infected women of childbearing potential. Indicators 7 to 11 were feasible in all 3 settings, and indicator 12 and 13 were exclusively feasible in Aruba. (Table 2)

#### Sample size

In Curaçao, indicator 6 ('maximally suppress viral load in HIV-infected women who wish to get pregnant') had a sample size of < 15 patients and was therefore rejected. All other indicators had large enough sample sizes for Curaçao. In Aruba only indicator 5 ('preconception counselling for all HIV-infected women') met the required sample size. In St Maarten, indicator 10 ('HIV-infected pregnant women who do not receive antiretroviral therapy antepartum') and indicator 11 ('scheduled caesarean section') could only be applied to 11 patients.

#### Inter-rater reliability

Indicator 12 ('counselling breastfeeding') scored a κ < 0.60 in all 3 settings. Indicator 5 ('pre-conception counselling') scored a Cohen's kappa coefficient κ < 0.60 in Aruba. Also, indicator 13 ('antiretroviral therapy in newborns') scored low inter-rater reliability for Aruba (κ = 0.11). Indicator 11 ('scheduled caesarean section') scored low inter-rater reliability for St Maarten (κ = 0.35). Indicator 9 ('discuss and provide antiretroviral therapy in all pregnant women') scored moderate inter-rater reliability in Curaçao (κ = 0.52).

#### Sensitivity to change

None of the potential indicators showed an overall high performance score. The performance of indicator 12 and 13 scored higher than 85% in St Maarten and indicator 12 scored higher than 85% in Aruba. The range between the highest and the lowest score of each indicator between the different settings was high for the indicators 5, 11, 12, and 13 (48%, 33%, 43% and 60% respectively).

#### Case mix stability

In St Maarten correction for multiparous women was necessary for indicator 12 ('counselling breastfeeding'). This indicator was more often measured in HIV-infected pregnant women with 2 or more pregnancies in the past than women with none or 1 pregnancy. No correction for type of health care insurance, age, or not born in the Dutch Caribbean was necessary for the other potential indicators.

## Discussion

This study shows the systematic development of quality indicators for HIV-infected (pregnant) women and their newborns in 3 different Dutch Caribbean settings; Curaçao, Aruba and St Maarten. Quality indicators are important as they provide insight in current care and they reveal areas that require further improvement of care. Thirteen indicators were selected and prioritized for the Dutch Caribbean: 4 concerning HIV screening in pregnant women, 2 concerning HIV-infected women, 6 concerning HIV-infected pregnant women and 1 concerning newborns of HIV-infected women. After testing the applicability of each potential indicator in practice only 4 indicators scored satisfactorily for Curaçao ('monitoring CD4-cell count', 'monitoring HIV-RNA levels', 'intrapartum antiretroviral therapy and infant prophylaxis if antepartum antiretroviral therapy was not received', 'scheduled caesarean delivery') and 3 for St Maarten ('monitoring CD4-cell count', 'monitoring HIV-RNA levels', 'discuss and provide combined antiretroviral therapy to all HIV-infected pregnant women'), whilst none for Aruba.

No consensus exists on how to best monitor the quality of care in HIV-infected pregnant women [22]. Most international studies report effectiveness of PMTCT services in a country or region by outcome or access to care (indicating the percentage of children infected or the percentage of HIV-infected pregnant women accessing PMTCT services) [11,22-27]. However, in order to reach the global goal of eliminating MTCT, monitoring the quality of the process of care seems to be as equally important as ensuring access especially in countries or regions that have already achieved high access to PMTCT services.

Several organizations and study groups have developed indicators regarding the care of HIV-infected pregnant women, mostly as part of a set of key indicators to measure the effectiveness of the implementation of a regional PMTCT program [28-35]. Five of such indicators, are process indicators, and show similarity to the quality indicators in our study namely indicator 1 ('HIV screening in all pregnant women'), indicator 5 ('preconception counselling'), indicator 9 ('antiretroviral therapy in all HIV-infected pregnant women'), indicator 12 ('counseling breastfeeding') and indicator 13 ('antiretroviral therapy in newborn'). Remarkably however, most of these well-known and internationally used indicators are currently not applicable in a Dutch Caribbean setting because they currently show lack of feasibility, inter-rater reliability or small sample sizes.

This study shows the importance of testing potential indicators for their applicability which has also been reported by others [21]. After assessing the applicability of each indicator in the 3 Dutch Caribbean settings, only 4 indicators could be satisfactorily tested in practice in Curaçao, 3 in St Maarten whilst none in Aruba. Firstly, applicability can only be tested if data are available to give information about the quality of care. In this study the indicators concerning HIV-infected women (indicator 5 and 6) and the indicator concerning newborns (indicator 13) showed low feasibility. For indicators with low feasibility it cannot be concluded that the limitation of data are due to improper data registration or incorrect implementation of the used guidelines. Proper surveillance, tracking systems or registration tools for collecting the necessary data should therefore be developed and made available before these quality indicators can be applied in the Dutch Caribbean setting.

Secondly, sizes of the samples on which the indicator operates have to be large enough. In small settings or in settings with low prevalence of HIV infection or with highly specific quality indicators accounting for only a specific proportion of the population, quality indicators cannot be used because of insufficient number of patients. This was evident in our study of the Aruban setting where only one indicator had a large enough sample size over a period of 10 years. Lowering the number of a sample size limits the statistical analyses necessary to develop the indicator and the statistical power when using the indicator in practice. The practical implication of limited statistical power is that patients and policymakers may not be able to properly identify quality problems in the clinical setting [36]. Given the limited usefulness of quality indicators in small populations it is worth considering additional approaches for judging quality of care in HIV infected (pregnant) women and their infants. The first approach would seem increment of sample size number by lengthening the time of the measurement, however this is not desirable since indicators should be dynamic over time. A second approach could be to provide more detailed information of the processes of care like review of complications [36] or case reporting. As the Caribbean region consists of multiple islands with relatively small populations like the Dutch Caribbean, the practical value of (specific) quality indicators for the region has to be questioned and a combination of methods of monitoring quality of care should be considered.

This study gives an overview of prenatal, delivery and child care in regard to PMTCT in 3 Dutch Caribbean islands. The study has led to identification of previously non-registered HIV-infected pregnancies and HIV-exposed children. It also created awareness of the quality of care regarding PMTCT and enhanced the possibilities for further discussion among health care professionals who are involved in planning and coordinating care. Although the applicability of some potential indicators was limited by overall small sample sizes and lack of feasibility one should note that the set of potential indicators had an overall low performance score. Only 2 indicators scored higher than 85%. Future initiatives aimed at improving the quality of care and eliminating the vertical transmission of HIV-infection in Curaçao, Aruba and, St Maarten should therefore be based on these study results.

Since access to HIV treatment has increased world-wide, a trend towards reporting on the quality of HIV treatment should be encouraged. To our knowledge this is one of the first reports on the quality of HIV treatment in the Caribbean.

Because pregnancies are currently not officially registered in the Dutch Caribbean, no dataset was available to test the 'screenings indicators' (indicator 1 to 4). This is a limitation of the study since the timely identification of HIV-infection by means of screening is essential in care and treatment of HIV-infected pregnant women. Also, no HIV rapid tests were available in the 3 settings, which may result in underreporting especially for those women presenting in labour with unknown HIV sero-status. Lack of proper screening may have influenced the applicability as well as the outcome of the quality of care provided, since reports show that patients who do not (timely) access proper care have worse outcomes [37-39]. Future initiatives to monitor the quality of care in HIV-infected pregnant women in the Dutch Caribbean should include the implementation of an official registration system for pregnancies or a prospective study in which screening patterns in pregnant women will be assessed. Another limitation of the study was that different clinical monitoring systems for HIV-infected patients were available in the 3 settings, none of them aimed at collecting data regarding the quality of care of HIV-infected pregnant women. Although we developed a unique Clinical Report Form for this study specific data may have been missed because data were collected retrospectively.

## Conclusion

In conclusion this is one of the first studies describing the systematic development of quality indicators for HIV-infected (pregnant) women. Our study shows the importance of applicability testing before implementing potential indicators even when the settings initially seem to be similar. In relatively small settings or settings with low prevalence, one should consider alternative approaches to monitor the quality of care; for example the reviewing of complications or case reporting. Furthermore, this study identifies areas for improvement in the collection of data and registration as well as areas for improvement in the quality of prenatal and delivery care in HIV-infected (pregnant) women and their newborns in the Dutch Caribbean.

## Competing interests

The authors declare that they have no competing interests.

## Authors' contributions

HH, LV, AJD designed the study, analyzed data and wrote the first draft. RV, FM, AD, GO, SK, IG and CS contributed to the interpretation of the data, have been critically revising the manuscript and have given final approval for publication. All authors read and approved the final manuscript.
